# Neurocardiogenic Syncope and Supraventricular Tachycardia in Association with a Rare Congenital Aortic Valve Abnormality

**DOI:** 10.1155/2016/8136079

**Published:** 2016-11-07

**Authors:** Yashwant Agrawal, Jagadeesh K. Kalavakunta, Vishal Gupta, William Lapenna

**Affiliations:** ^1^Department of Internal Medicine/Pediatrics, Western Michigan University Homer Stryker School of Medicine, Kalamazoo, MI, USA; ^2^Department of Cardiology, Michigan State University/Borgess Medical Center, Kalamazoo, MI, USA

## Abstract

We report a case of a 26-year-old woman who presented with multiple episodes of syncope over a five-months period of time. Transthoracic echocardiogram had shown a normal functioning quadricuspid aortic valve (QAV) which was also confirmed on a transesophageal echocardiogram. Computed tomographic angiography of heart and coronary arteries showed the QAV with equal size of all aortic cusps and normal coronary arteries. Intermittent chest pain and palpitations warranted an exercise stress test. The stress test revealed normal aerobic exertion, with achievement of 101% of maximal peak heart rate. However, during peak stress, we noted a drop in her blood pressure significantly resulting in dizziness. No arrhythmias were noted during the stress test. With recurrent syncope episodes and palpitations, Holter monitoring was done, revealing supraventricular tachycardia (SVT). We discuss current available literature and coassociations with QAV. New association of QAV with SVT needs further analysis.

## 1. Case Report

**Figure 1:**
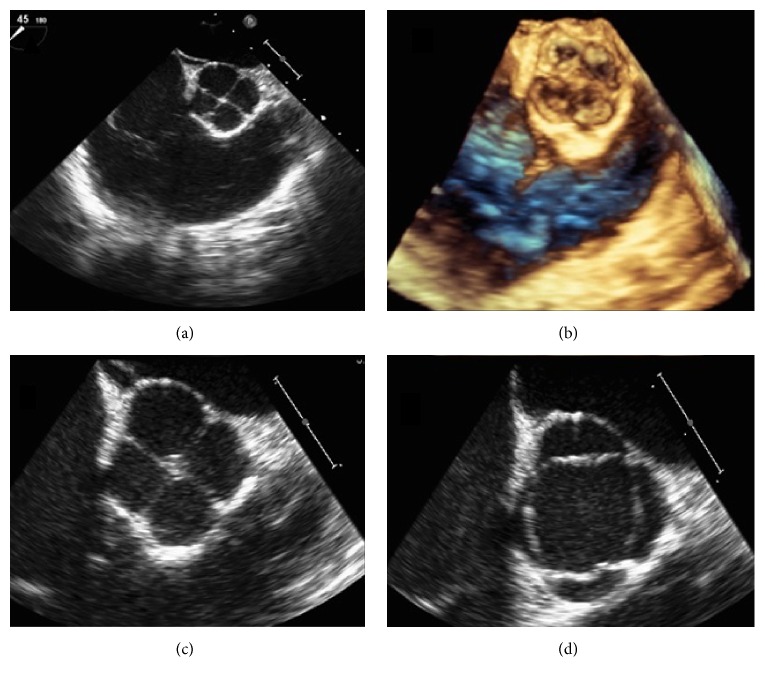
Transesophageal echocardiogram short axis view of the aortic valve revealing QAV. (a) 2-dimensional and (b) 3-dimensional view of the same image. (c) and (d) showing the zoomed view of the short axis QAV in diastole and systole, respectively.

**Figure 2:**
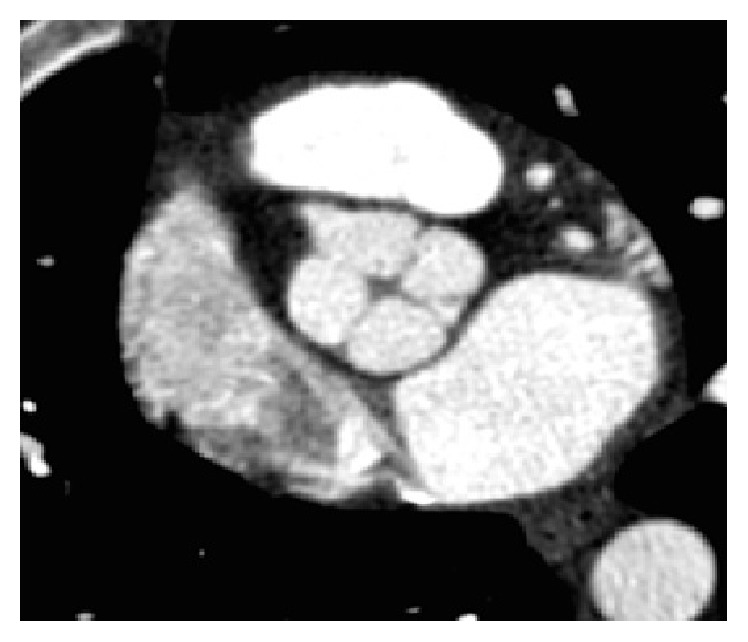
Heart CTA showing QAV.

A 26-year-old woman presented to the emergency department (ED) with complaints of syncope and palpitations occurring at her workplace, when she was lifting a 15-pound object. Syncopal episode was preceded by “color appearing abnormal,” as noted by coworker, and lasted approximately five minutes at which time she regained consciousness without a postictal state. Physical examination including orthostatic vitals was normal. Laboratory data and electrocardiogram (EKG) were unremarkable. Transthoracic echocardiogram (TTE) revealed a possible quadricuspid aortic valve (QAV). Transesophageal echocardiogram (TEE) confirmed a QAV without stenosis or regurgitation (Figure 1). No aortic wall or coronary artery abnormalities were detected. Computed tomography angiography (CTA) of the heart showed a QAV with equal size of all aortic cusps and normal coronary arteries including no congenital abnormalities related to their origin or in their anatomy (Figure 2). The QAV was felt to be a benign pathology, with no stenosis or regurgitation and patient was discharged. Patient presented to the ED 3 months later with multiple syncopal episodes, this time accompanied by sharp chest pain lasting for few seconds. Episodes were reportedly brought upon by physical activity. Physical examination and repeat laboratory data were unremarkable. Electrocardiogram (ECG) revealed normal sinus rhythm. Exercise stress test revealed excellent aerobic capacity of 20 minutes. However, she felt dizzy at the peak effort and stress (stage 6), with blood pressure reading of 116/52, a significant drop from her initial reading of 155/72 mm Hg. Her heart increased from 166 bpm to 193 bpm. She was discharged with a 48-hour Holter monitor in place. Holter evaluation revealed a single episode of supraventricular tachycardia (SVT), lasting 39 seconds at a rate of 155 beats per minute, though she was asymptomatic during this event. The SVT episode occurred in the morning when she was lying in bed. She was seen in the cardiology office and started on 25 mg atenolol for the SVT. On this treatment, she has done well, remaining asymptomatic without further syncopal episodes as noted at consistent outpatient follow-up visits. With patient's resolution of symptoms, a head upright tilt table testing was not done but was considered. Also, firm diagnosis of SVT leading to patient's syncope could not be made.

## 2. Discussion

QAV is a rare congenital aortic valve abnormality, the true incidence of which is not known. Most cases were previously identified on autopsies, with 0.008% incidence on 25,644 autopsy studies [1]. QAV has been subdivided into 7 groups based on size of the aortic leaflets. Majority are type A (4 equal cusps), B (3 equal cusps with 1 smaller cusp), or C (2 equal larger and 2 equal smaller cusps) [2].

QAV mostly occurs as an isolated finding [1, 3]. Its association with other cardiac anomalies is of great importance (18.3%). An increased aberration of coronary arteries was seen in 10% of cases [4] who can present as sudden cardiac death. Our patient had an isolated QAV. The most frequently reported functional problem with a QAV is aortic insufficiency. It was described in 75% of the cases by Tutarel [4] and 56% of the cases by Janssens et al. [3]. A normal functioning QAV was seen in only 16–25% in these case studies.

There has been one reported case of congenital complete heart block with QAV [5]. There are no other reported cases of arrhythmogenic activity in association with QAV. The symptoms resolved after the patient was started on metoprolol which corelates with her symptoms being most likely from SVT. However, QAV was functioning normally; hence there is a likelihood of the association between QAV and SVT being causal. The fact that the patient was lying in bed during the SVT recorded on Holter monitor explains her being asymptomatic during the episode. With no recommendation per valvular guidelines for monitoring QAV, physician awareness of associated anomalies and further evaluation of the same anomalies can be life-saving for patients. In-depth history and thorough physical examination with focus on development of new murmur can detect dysfunctional valve early.
